# Voltage-Sensing Domain of the Third Repeat of Human Skeletal Muscle NaV1.4 Channel As a New Target for Spider Gating Modifier Toxins

**DOI:** 10.32607/actanaturae.11279

**Published:** 2021

**Authors:** M. Yu. Myshkin, A. S. Paramonov, D. S. Kulbatskii, E. A. Surkova, A. A. Berkut, A. A. Vassilevski, E. N. Lyukmanova, M. P. Kirpichnikov, Z. O. Shenkarev

**Affiliations:** Shemyakin-Ovchinnikov Institute of Bioorganic Chemistry, Russian Academy of Sciences, Moscow, 117997 Russia; Biological Faculty, Lomonosov Moscow State University, Moscow, 119234 Russia

**Keywords:** cell-free protein synthesis, ligand–receptor interaction, NMR spectroscopy, sodium channels, gating modifier toxins

## Abstract

Voltage-gated sodium channels (NaV) have a modular architecture and contain five membrane domains. The central pore domain is responsible for ion conduction and contains a selectivity filter, while the four peripheral voltage-sensing domains (VSD-I/IV) are responsible for activation and rapid inactivation of the channel. “Gating modifier” toxins from arthropod venoms interact with VSDs, influencing the activation and/or inactivation of the channel, and may serve as prototypes of new drugs for the treatment of various channelopathies and pain syndromes. The toxin-binding sites located on VSD-I, II and IV of mammalian NaV channels have been previously described. In this work, using the example of the Hm-3 toxin from the crab spider *Heriaeus melloteei*, we showed the presence of a toxin-binding site on VSD-III of the human skeletal muscle NaV1.4 channel. A developed cell-free protein synthesis system provided milligram quantities of isolated (separated from the channel) VSD-III and its 15N-labeled analogue. The interactions between VSD-III and Hm-3 were studied by NMR spectroscopy in the membrane-like environment of DPC/LDAO (1 : 1) micelles. Hm-3 has a relatively high affinity to VSD-III (dissociation constant of the complex Kd ~6 μM), comparable to the affinity to VSD‑I and exceeding the affinity to VSD-II. Within the complex, the positively charged Lys25 and Lys28 residues of the toxin probably interact with the S1–S2 extracellular loop of VSD-III. The Hm-3 molecule also contacts the lipid bilayer surrounding the channel.

## INTRODUCTION

**Fig. 1:**
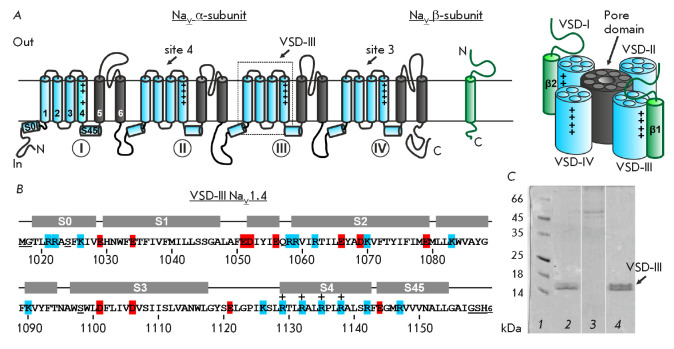
(*A*) Spatial organization of eukaryotic NaV channels in the membrane. (*B*) Amino acid sequence of VSD-III used in the study. Artificially introduced residues are underlined. The secondary structure is presented according to the known spatial structure of the channel [6] (PDB ID: 6AGF). The negatively and positively charged residues are highlighted in red and blue, respectively. Positively charged residues in the voltage sensor S4 helix are marked by a “+” sign. (*C*) Purification of VSD-III by Ni^2+^-affinity chromatography. Lanes: *1 *–molecular weight marker; *2 *–solubilized RM pellet;* 3 *–column wash; and *4 *–elution with 500 mM imidazole. Molecular weight of VSD-III: 16.3 kDa

Voltage-gated Na^+^-channels (Na_V_) are transmembrane (TM) proteins responsible for the ascending phase of the action potential in excitable cells. These channels consist of a pore-forming α-subunit with which regulatory β-subunits are associated (*Fig. 1A*). The α-subunit includes four homologous repeats (I–IV), each of those containing a voltage-sensing domain (VSD, TM segments S1–S4) and S5–S6 segments that form the pore of the channel [1]. The β-subunits have one TM segment and an extracellular immunoglobulin domain [2]. The human genome contains 10 genes encoding the α-subunits of Na_V_ and four genes encoding β-subunits. The Na_V_1.4 channel is expressed in skeletal muscle, and mutations in its α-subunit gene (*SCN4A*) lead to a number of congenital disorders of the musculoskeletal system, such as myotonia, paramyotonia, hyperkalemic and hypokalemic periodic paralysis, myasthenia gravis, and myopathy [3].

Na_V_s are targets for many neurotoxins from different organisms. At least eight receptor-binding sites for toxins have been identified in the VSD and the pore of the channel [4]. In the extracellular loops of VSDs of repeats II and IV, two canonical binding sites for spider and scorpion toxins were identified (*Fig. 1A*) [5]. Toxins acting on VSD-IV (site 3) inhibit channel inactivation, and toxins acting on VSD-II (site 4) (e.g., “gating modifier” toxins of spiders) affect channel activation [5]. Although the extracellular interfaces of VSD-I and III can be partially closed by the immunoglobulin domains of the β-subunits [6, 7], these domains, involved in channel activation, may also contain toxin-binding sites available in some pathophysiological conditions. Thus, it was shown [8] that some toxins inhibit the activation of the chimeric K_V_2.1 channel containing S3–4 loops from VSD-I or III of the Na_V_1.2 channel and do not inhibit the original K_V_2.1 channel. The search for the binding site of neurotoxins in eukaryotic channels by site-directed mutagenesis is difficult, since the α-subunit of Na_V_ contains four VSDs, each of which can take part in the formation of a response to the toxin’s action.

Earlier, we showed that the extracellular loop S3–S4 of VSD-I of the human Na_V_1.4 channel is the main binding site for the Hm-3 toxin from the venom of the spider *Heriaeus melloteei *[9]. In addition, Hm-3 interacts with the S1–S2 extracellular loop of VSD-II, but with a much lower affinity [10]. The Hm-3 toxin consists of 35 amino acid residues and has a charge of +4 at neutral pH. The secondary structure of Hm-3 includes several β-turns and a β-hairpin formed by Cys23–Cys34 residues. The spatial structure of Hm-3 is stabilized by three disulfide bonds, which form the so-called “cystine knot” [11]. Several aromatic residues form a hydrophobic cluster on the surface of Hm-3; therefore, like other “gating modifier” spider toxins, Hm-3 has an affinity for membranes [11] and, apparently, attacks the VSDs from the membrane-bound state. Toxins that belong to this family are interesting not only as tools for the structural and functional study of Na_V_, but they can also serve as prototypes for new drugs. For example, Hm-3 can block aberrant leakage currents (ω-currents) arising in the Na_V_1.4 channel with mutations in VSD-I and II, leading to the development of periodic paralysis [9, 10].

In this work, using the Hm-3 toxin as an example, we have shown for the first time that a toxin-binding site is present in VSD-III of the human Na_V_1.4 channel. In order to do this, we used an alternative approach based on the production of a recombinant isolated (separated from the channel) VSD and an analysis of the binding sites by NMR spectroscopy. Several works have demonstrated that it is possible to perform structural NMR studies of isolated VSDs [12] and their complexes with toxins [9, 10].

## MATERIALS AND METHODS

Isolated VSD-III (residues 1019–1157, *Fig. 1B*) was obtained using a dialysis-type conjugated cell-free synthesis system based on the S30 extract from* Escherichia coli *using protocols developed for other VSDs [9, 10]. The genetic construct for synthesizing VSD-III with the C-terminal His6-tag was cloned into the pIVEX2.3d plasmid vector, which provides a high efficiency in cell-free synthesis. The VSD-III sequence contains two Cys residues that are not involved in the formation of disulfide bonds. To reduce the tendency towards aggregation of the recombinant VSD-III, these residues were replaced by Ser (*Fig. 1B*, underlined). Cell-free synthesis was performed without adding membrane-mimicking components to the reaction mixture (RM). In this case, the synthesized VSD-III accumulated in the form of a precipitate with a purity of more than 90% (*Fig. 1C*). A 15N-labeled analogue of VSD-III was synthesized using a 15N isotope-enriched mixture of 16 amino acids (Cortecnet, Les Ulis, France) obtained from algae and the individual 15N-labeled amino acids Asn, Gln, and Trp. Cysteine was not added to the synthesis reaction, since the VSD-III variant used in this work did not contain that amino acid. The yields of unlabeled and ^15^N-labeled VSD-III samples were 0.5 and 0.35 mg per 1 mL of RM, respectively. For NMR studies, the precipitate containing the synthesized VSD-III was dissolved in a 10% dodecylphosphocholine (DPC) solution, purified by Ni^2+^ affinity chromatography in the presence of 0.5% DPC (*Fig. 1C*), and transferred to the target buffer (20 mM Tris-Ac, pH 5.5), and the N,N-dimethyldodecylamine-N-oxide (LDAO) detergent was added to a 1: 1 molar ratio with DPC. Previously, mixed DPC/LDAO micelles were used as a membrane-mimicking medium to study complexes of VSD-I and II with the Hm-3 toxin [9, 10]. Detergent concentrations were monitored by 1D 1H NMR spectra. The NMR spectra were recorded on an AVANCE III 800 spectrometer (Bruker).

## RESULTS AND DISCUSSION

**Fig. 2:**
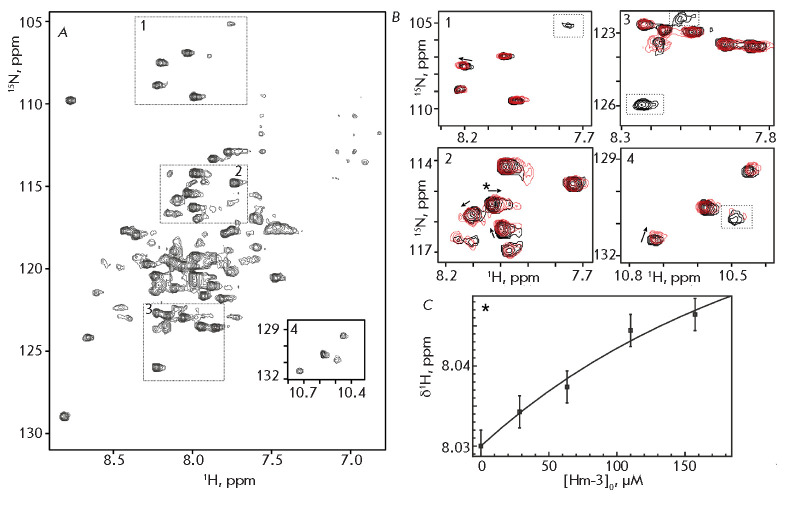
The NMR study of 15N-labelled VSD-III interaction with non-labelled Hm-3. (*A*) The 2D 1H,15N-TROSY spectrum of 43 rKM VSD-III in DPC/LDAO micelles (45/45 mM, 800 MHz, pH 5.5, 45°C). (*B*) Overlay of the VSD-III spectral fragments, before (black) and after addition of 160 rKM Hm-3 (red). Arrows indicate the direction of the changes in signal position. Dashed lines indicate the signals that disappear after the addition of Hm-3. (*C*) Changes in 1H chemical shift of the HN signal at 8.03/115.4 ppm (marked by an asterisk on panel *B2*) approximated by the equation describing binding in the presence of an excess of detergent micelles

The general appearance of the 2D ^1^H,^15^N correlation NMR spectrum of VSD-III (*Fig. 2A*) corresponded to the spectra of VSD-I and II obtained earlier [9, 10]. The observed small dispersion of 1HN signals is characteristic of helical TM proteins. However, the spectrum contained no more than 90 signals of backbone HN groups out of the 130–140 expected signals. In the corresponding spectral regions, six of the eight HN signals of the Gly residues and four of the five HNε1 signals of the side chains of the Trp residues are presented. The absence of some signals in the spectrum, as well as the inhomogeneous intensity and half-width of the observed signals, is indicative of a conformational exchange in the μs–s range. These processes are probably associated with the plasticity of the VSD-III structure and the dynamics of contacts between TM helices. The observed signal-broadening did not allow us to obtain assignment of the VSD-III NMR signals; therefore, the interaction with Hm-3 was studied qualitatively, without mapping of the binding site in the VSD.

Samples of unlabeled and ^15^N-labeled Hm-3 were obtained using recombinant production in *E. coli *cells [9, 11]. To study the interaction of VSD-III with Hm-3, unlabeled Hm-3 was added stepwise to a sample of ^15^N-labeled VSD in DPC/LDAO micelles to a 1 : 4 molar ratio of VSD/toxin. Detergent concentration was kept constant to prevent changes in the toxin distribution between the water phase and the micelles. According to the previously obtained data on the interaction of Hm-3 with DPC/LDAO micelles [9], ~97% of toxin molecules bound to micelles under the experimental conditions. After addition of the toxin, changes in the chemical shifts and amplitudes of some signals were observed in the spectrum of VSD-III (*Fig. 2B*). These changes were an indication that the VSD–toxin interaction was specific. The reversible process of formation– dissociation of the VSD/Hm-3 complex has a characteristic time in the μs–s range, and for different VSD signals this exchange process is either fast or intermediate (on the NMR time scale). The dissociation constant of the complex was determined by approximating the dependence of the chemical shift of the VSD-III signals on the Hm-3 concentration (*Fig. 2B*), taking into account the contribution of the Hm-3/ micelle interaction [9]. The obtained value (5.8 Ѓ} 3.8 μM) corresponded to the dissociation constant of the VSD-I/Hm-3 complex (6.2 ± 0.6 μM) [9] and was lower than the value for the complex with VSD-II (~11 μM) [10], which indicates stronger interaction of the toxin with VSD-I and VSD-III.

**Fig. 3:**
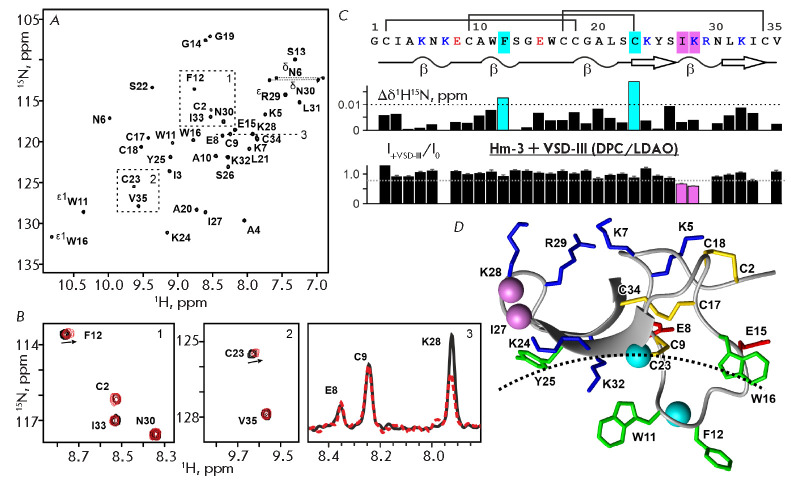
The NMR study of interaction between 15N-labelled Hm-3 and non-labelled VSD-III. (*A*) The 2D 1H,15N-HSQC spectrum of 30 rKM Hm-3 in DPC/LDAO micelles (45/45 mM, 800 MHz, pH 5.5, 45°C). (*B*) Overlay of the Hm-3 spectra fragments, before (black) and after addition of 40 rKM VSD-III (red, the final VSD-III/Hm-3 ratio is 3 : 2). Arrows indicate the direction of changes in the signal position. Panel *B3 *depicts the selective decrease in Lys28 signal intensity after addition of VSD-III. (*C, D*) The primary, secondary, and spatial structures of Hm-3 toxin [11] (PDB ID: 2MQU), and the changes in the chemical shifts and intensities of Hm-3 signals after addition of VSD-III. The positively and negatively charged residues and disulphide bridges are shown. Residues whose signals undergo significant changes are highlighted (*C*) and marked by balls (*D*). The dashed line represents the detergent micelle surface [9]

Back titration, when unlabeled VSD-III was added to a sample of ^15^N-labeled Hm-3, showed that the positively charged residues Lys25 and Lys28 located in the β-hairpin of the toxin, as well as the Phe12 residue buried in the hydrophobic region of the micelle, are involved in the formation of a complex with VSD-III (*Fig. 3*). This binding site coincides with the sites responsible for the interaction of Hm-3 with VSD-I and II [9, 10]. In the course of these earlier studies, it was shown that the pair of charged Hm-3 residues (Lys25 and Lys28) can specifically interact with helical motifs containing two negatively charged residues (Asp or Glu) separated by two or three uncharged residues. In the VSD-III sequence, such motifs are found only in the S1–S2 extracellular loop and in the TM portion of the S2 helix (*Fig. 1B*). However, according to the wellknown spatial structure of the human NaV1.4 channel [6], the Glu1066 and Asp1069 residues are located deep in the TM portion of the S2 helix and their side chains are turned inside the VSD-III molecule. Taking into account the amphipathic properties of Hm-3 [11], we assume that the toxin cannot penetrate deep into the membrane and interact with these residues. Meanwhile, the side chains of the Glu1051, Asp1052, and Glu1056 residues located in the S1–S2 loop region are accessible to the solvent and can interact with the Hm-3 molecule bound to the membrane surface.

On the contrary, another extracellular loop of VSD-III, S3–S4, contains a single negatively charged residue Glu1121 and probably cannot act as a binding site for the Hm-3 toxin. This is consistent with the results of a previous study of the K_V_2.1 chimeric channel containing the loop S3–S4 transplanted from the VSD-III channel Na_V_1.4, during which no significant interaction with the Hm-3 toxin was revealed [9]. Thus, the data obtained indicate that the extracellular loop S1–S2 of the VSD-III of the human Na_V_1.4 channel contains a site capable of interacting with “gating modifier” spider toxins. It should be noted that the study of toxin-binding sites located at the S1–2 region of the VSD of Na_V_ channels using chimeric channels is apparently impossible. Attempts to transplant S1–S2 loops from various channels into K_V_2.1 resulted in nonfunctional chimeras [13].

The system of cell-free synthesis of VSD-III developed in this work will make it possible to further investigate the interaction between the domain and other toxins and can also be used for screening drug prototypes that selectively interact with VSD-III. The proposed method for NMR study of Na_V_ pharmacology has an advantage over methods based on the study of chimeric channels, since it allows one to map the toxin residues important for interaction with voltage-sensing domains and study toxin-binding sites that are located not only in the S3–S4, but also in the S1–S2 loop.

## Abbreviations

DPC: dodecylphosphocholine
LDAO: n-dodecyl-N,N-dimethylamine-N-oxide
NaV: voltage- gated sodium channel
VSD: voltage-sensing domain
VSD-III: voltage-sensing domain from the third repeat of α-subunit of human NaV1.4 channel
TM: transmembrane
RM: reaction mixture
